# Beyond Formal Access: Organizational Context, Working From Home, and Work–Family Conflict of Men and Women in European Workplaces

**DOI:** 10.1007/s11205-018-1993-1

**Published:** 2018-10-05

**Authors:** Tanja van der Lippe, Zoltán Lippényi

**Affiliations:** Utrecht University, Utrecht, The Netherlands

**Keywords:** Working from home, Work–family conflict, Organizational context, Gender, Multi-level

## Abstract

Working from home has become engraved in modern working life. Although advocated as a solution to combine work with family life, surprisingly little empirical evidence supports that it decreases work–family conflict. In this paper we examine the role of a supportive organizational context in making working from home facilitate the combination of work and family. Specifically, we address to what extent perceptions of managerial support, ideal worker culture, as well as the number of colleagues working from home influence how working from home relates to work–family conflict. By providing insight in the role of the organizational context, we move beyond existing research in its individualistic focus on the experience of the work–family interface. We explicitly address gender differences since women experience more work–family conflict than men. We use a unique, multilevel organizational survey, the European Sustainable Workforce Survey conducted in 259 organizations, 869 teams and 11,011 employees in nine countries (Bulgaria, Finland, Germany, Hungary, Netherlands, Portugal, Spain, Sweden, United Kingdom). Results show that an ideal worker culture amplifies the increase in work family conflict due to working from home, but equally for men and women. On the other hand, women are more sensitive to the proportion of colleagues working from home, and the more colleagues are working from home the less conflict they experience.

**Electronic supplementary material:**

The online version of this article (10.1007/s11205-018-1993-1) contains supplementary material, which is available to authorized users.

## Introduction

Working from home is now entrenched in modern working life. In 1996, only 20% of US companies had working from home arrangements, but by 2016 this had grown to 60% (SHRM 2016). In Europe, it was estimated that approximately one out of eight workers work from home at least several times a month on average across the EU 28 countries (Chung 2018). Against the backdrop of a growing number of dual-earner couples, working from home was touted in the 1980s and 1990s as a cost-effective option for having less work–family conflict (Avery and Zabel 2011). However, there is puzzlingly little empirical evidence which suggests that working from home could be an effective way to mitigate work–family conflict (Allen et al. 2015a; Golden et al. 2006; Kossek et al. 2006). Human resource management theory argues that work flexibility, such as the opportunity of working from home benefits employees by giving them more discretion in combining work and family tasks (Appelbaum 2000; Whitener 2001; Ortega 2009). However, the potential downside is that flexible working is coupled to, often implicit, expectations of high effort and commitment, offsetting potential gains for less work–family conflict (Godard 2001; Wright and Raley 2008; Jensen et al. 2013; Kelliher and Anderson 2010; Lott and Chung 2016; see also, Chung and Van der Horst, in this issue; Chung and Van der Lippe, in this issue (introduction)). If these insights are correct, the capacity of working from home to alleviate work–family conflict may depend on how supportive the organizational context is for the work–family needs of workers *beyond* providing formal access (cf. Lewis 2001).

There is evidence that a supportive organizational context is important to alleviate work-life conflict (cf. Den Dulk et al. 2016; Chung, in this issue), but a number of scholars indicated that there is void of research studying how cultural-normative organizational contexts are of influence on family-friendly policies and employee outcomes (Allen et al. 2015b; Kossek et al. 2006; Den Dulk et al. 2016). The present paper fills this gap by providing insight in how the organizational context shapes the impact of working from home on work–family conflict. Specifically, we investigate the role of the ideal worker culture (Kelly et al. 2010), managerial work-life support, and co-worker’s engagement in working from home. By studying the role of culture, support and co-worker behaviour, our study answers recent efforts to contextualize work–family research, “abandoning the tight focus on individuals’ experience of the work–family interface” (Williams et al. 2016, p. 521). In this contribution working from home refers to working at or from home during (at least part of) the employees’ contractual working hours (Felstead and Jewson 2000; Peters and Van der Lippe 2007). In most cases information or communication technology is used to interact with others both within and external to the central office (Allen et al. 2015a).

Gender differences in work–family conflict are paramount (Duxbury and Higgins 1991; Hagqvist et al. 2017; See also, the introduction (Chung and Van der Lippe in this issue), as women experience greater tensions between work and family life than men (Crompton 2002). Research focusing on gender differences in utilizing work–family benefits, such as working from home, showed that men and women judge their utility based on different criteria: men considered work–family benefits useful when they believed it benefitted job performance, while women tended to judge their effectiveness based on expected reduction of work–family conflict (Sprung et al. 2015). Gender-biased selection may also account for underutilizing benefits: especially men may refrain from utilizing working from home for the fear of negative career consequences (Greenhaus and Kossek 2014). Findings on gender differences in the impact of work-life benefits on work-life conflict are, however, scattered. Hammer et al. (2007) showed that using family friendly benefits actually leads to increased perceptions of conflict for women because they use this opportunity to take on more family responsibilities, but our knowledge is very limited whether a supportive organizational context impacts the gains of men and women from working from home differently. Existing research unearthed gender differences in the usefulness of informal organizational support, but it primarily focused on gender similarity between the worker and supervisor (e.g., Foley et al. 2006), and not on gains from formal support, such as working from home. In this contribution we therefore also examine the role of gender in the relation between working from home and work–family conflict. Given the fact that women experience more work–family conflict than men, working from home might help to gain more understanding in why this is the case.

Despite the recent plea of De Menezes and Kelliher (2011) for multilevel designs to study how flexible working arrangements influence employee outcomes, current research lacks such data. Most studies in the literature on working from home either used worker-level data from a single organization limiting generalizability beyond a given organizational context and the potential to study organizational differences, or data at the organizational level that do not allow to assess how working from home impacts individual outcomes, such as work–family conflict, or study gender differences (Bloom et al. 2014; Rousseau 2011). Our study makes use of a newly collected, large-scale survey of 259 organizations, 869 work units, and 11,011 employees in multiple economic sectors in the UK, Germany, Hungary, Bulgaria, Spain, Sweden, Finland, the Netherlands, and Portugal (Van der Lippe et al. 2016). This multi-level dataset enables us to study the returns to working from home for workers across different organizational contexts. In line with multilevel theorists, we argue that work–family conflict is more than adding up individual efficiencies from work (Kozlowski and Klein 2000).

To sum up, we try to make the following three contributions: first, in understanding the consequences of working from home for family life, we take into account the workplace context. Second, we pay attention to the gendered effects of working from home and potential differences in how culture, support, and behaviors in the work context impacts the work–family conflict of men and women. Third, we use a large-scale multilevel study in diverse organizational contexts which makes it possible to study how differences among employees in teams in organizations influence work–family conflict.

## Theory and Hypotheses

### Working From Home and Work–Family Conflict

There are different reasons why organizations offer working from home, but a main rationale is to foster employee work-life balance and well-being (Been et al. 2016). Bringing work to home, however, could lead to the deconstruction of boundaries and conflict between work and private life (Glass and Noonan 2016). The implications of working from home for work-life conflict are therefore far from obvious. There are two lines of thoughts.

On the one hand, working from home reduces work–family conflict because it provides employees control over the scheduling of their workdays. Being able to choose a location for work allows employees to use time more efficiently and to schedule various activities in a way that suits the employee’s situation (Gajendran and Harrison 2007; Parasuraman and Greenhaus 2002), leading to less conflict. Employees who work from home frequently use electronic communication, bringing time efficiencies: it enables them to stay in touch with work, also on schedules that differ from colleagues or customers (Ten Brummelhuis et al. 2012). Furthermore, working from home could also save time because telecommuters can cut down on commuting that cannot be used for work or family activities (Hill et al. 2003; Kossek and Thompson 2016). For these reasons, working from home is linked with more control of time and higher levels of autonomy (Madsen 2003) and hence viewed as a measure to decrease work–family conflict.

There are theoretical views, however, that working from home has the opposite effect on work-life conflict. Assuming that employees have multiple roles (e.g., of employee, spouse and parent) that draw on the same scarce resources, working from home may interfere with performing responsibilities in the home domain such as taking care of domestic duties and leads to conflict (Peters and Van der Lippe 2007; Voorpostel 2014; Kossek and Thompson 2016). In addition, working from home increases the permeability of boundaries between work and non-work domains because the physical boundaries between the two contexts are eliminated (Shamir and Salomon 1985; Guest 2002). The loss of a clear physical boundary may result in thoughts and emotions from the work sphere more easily spilling over into the household domain, leading to work–family conflict (Clark 2000), and may even lead to extra domestic and care work (Kim in this issue; Kurowska in this issue). Working from home may also allow workers to work longer than they would have otherwise, increasing their work capacity but also increasing the probability for workers to experience more conflict due to the expansion of both spheres. Finally, efficiently working from home also requires greater self-control from workers, which can be a serious pitfall that possibly leads to overtime work and greater work–home interference (Sullivan and Lewis 2001). The strain related to these aspects of working from home might then result in more work–family conflict (Golden et al. 2006).

Overall, there seems to be more indication that working from home leads to work–family conflict than the opposite (Allen et al. 2015a). Studies showed that telecommuters work longer and experience more time pressure (Peters and Van der Lippe 2007; Glass and Noonan 2016), and instead of facilitating balance, working from home leads to interference between family and work roles (Peters et al. 2009; Hill et al. 2003). Duxbury and Higgins (1991) also found that users of telecommuting were more likely to experience stress and work–family interference. Similarly, flexible work schedules may cause stress owing to constantly changing schedules that result in a lack of structure of daily program (Tausig and Fenwick 2001). Finally, there is also evidence that electronic devices frequently used by teleworkers, such as mobile devices, blur the distinctions between the public and private domains of life (Green 2002; Mazmanian et al. 2013). For instance, Jarvenpaa and Lang (2005) showed that smartphone users reported increased work pressure and the inability to separate and keep distance from work. This might be because boundary management is more difficult for workers who are able to work from home, as the blurring of role boundaries will likely occur more when working from home is combined with smartphone use or other forms of continuous connection. In sum, based on evidence which is more in favor of a negative than a positive effect of working from home, we *hypothesize* that working from home leads to work–family conflict (H1).

### The Role of the Organizational Context

The degree to which an organization can be viewed as instilling a family-supportive work context is rooted in its work–family culture. Work–family culture is defined as “the shared assumptions, beliefs, and values regarding the way in which an organization supports and values the integration of employees’ work and family” (Thompson et al. 1999, p. 394). Allen (2001) describes a supportive organizational culture as one that acknowledges and is supportive of employees’ family and personal situations, and promotes flexibility, tolerance and support for family needs and obligations. These organizations are unlike those that have an ideal worker culture (Kelly et al. 2010, Williams 2000), theoretically establishing an employee’s commitment, dedication and value to the organization contingent on the number of hours they are in the office and whether they make their work responsibilities a top priority. Supportive work–family culture produces norms that respect employees’ personal and family time, and encourage use of work–family benefits, such as working from home. This is in contrast to the ideal worker culture where employees are expected to work long hours and arrange their responsibilities around their paid work themselves. Also the take up of flexible working arrangements, especially to facilitate work/family demands may not be supported or even penalized (Williams et al. 2013; Chung in this issue).

The perceptions of a work–family supportive context decreases work–family conflict, above and beyond the number of family-friendly benefits available by the organization (Allen 2001). Individuals perceiving their organization to be supportive of family needs may feel more comfortable devoting time and energy to their family and personal life without fearing the negative career consequences. They may also feel less pressured to invest themselves completely in their work role at the expense of their family. Perceived managerial work-life support leads to less work–family conflict as well, as it is easier to discuss conflicting issues and more likely to receive empathy from a supervisor who is supportive of work-life issues, instead of one who views these as a private matter (Den Dulk et al. 2016; Major et al. 2008). We thus argue that a work–family supportive context may also be influential to what extent working from home causes work–family conflict. Specifically, working from home may be less stressful and conflicting when managers are perceived to be more supportive for work–family needs, because perceived support acts as a resource (cf. Bakker and Demerouti 2007) providing latitude to cope with boundary conflicts arising from working from home. However, if an ideal worker norm prevails in the organization, and the work culture is performance-oriented, demanding greater effort and time from the worker, users of work–family benefits may fear backlash and stigmatization from colleagues and supervisors, which serves as a stressor that contributes to work–family conflict (Allen et al. 2013). In these workplaces, that place emphasis on work targets and performance, less “face time” with supervisors and managers arguably induces more fear for job and careers than in organizations that put less emphasis on the private needs of workers (Kelly et al. 2010). We formulate the following hypotheses: higher levels of managerial work–family support will buffer the negative effect of working from home on work–family conflict (H2a) and the ideal worker culture will increase the positive effect of working from home on work–family conflict (H2b).

Next to perceptions of the culture, co-workers’ engagement in working from home may also mitigate the work–family conflict of working from home. In work environments, the prevalence of a behavior has consequences for perceptions of normativity of the behavior (cf. Ajzen 1991). In addition, workers frequently practicing a certain activity or behavior creates ‘community of practice’ of shared expertise, knowledge, and understanding of problems and solutions associated with the behavior (Wenger 1998). From a normative perspective, co-workers engagement in working from home at the workplace generate beliefs that it is acceptable to utilize this benefit. This reduces feelings among teleworkers that they are singled out. In contrast, those workers who are working from home in an environment where very few co-workers do, may experience isolation (Golden et al. 2008), reducing perceptions of work support. Co-workers engagement in teleworking is likely to contribute to the development of a ‘community of practice’ around teleworking. That is, when many workers utilize working from home, the organization and co-workers are likely to provide support for efficient teleworking, for instance with portable devices or online working platforms and support for online working groups (cf. Watson-Manheim et al. 2002), helping these workers to work efficiently and avoid stress. In addition, in a context where many co-workers work from home, co-workers are more likely to be understanding of the potential conflicting situations, so when one encounters such situations, they are better equipped to deal with them. Based on these arguments, we expect that the more co-workers engage in working from home, the less negative the effect of working from home on work–family conflict will be (H2c).

### Differences Between Men and Women

Although a number of men and women adhere to egalitarian values nowadays, societal norms often dictate that men should invest more in the work domain, whereas women should invest more in the family domain. These opinions together with traditional gender role attitudes still greatly influence how men and women are able to manage work and family roles (Van der Lippe 1994; Treas and Drobnic 2010; Hagqvist 2016). To what extent working from home weakens the boundary between work and home will likely work out differently for men and women. Several authors (Sullivan and Lewis 2001; Kim in this issue) argued that women who work from home are able to fulfil their domestic role better and manage their work and family obligation more to their satisfaction, but that comes at the expense of higher perceived work–family conflict (see also Hilbrecht et al. 2008). Male teleworkers on the other hand, import industrial time into the home, but they do not let domestic obligations interfere with their work tasks, although the extent to which this happens may depend on the country contexts (Kurowska in this issue). They can do this because they are rarely the primary carer and/or the person responsible for household domestic work (Van der Lippe 1994). We therefore expect that the negative effect of working from home on work–family conflict is stronger for women than for men (H3).

Do telecommuting men and women experience work–family conflict differently in more family-supportive work contexts? We argue that telecommuting women would profit more from a family supportive organizational culture, because of the following reasons. First of all, given that the family domain is typically more salient for women than for men, working women are more sensitive to a supportive organizational context than men (Rupert et al. 2012; Thompson and Cavallaro 2007). Blanch and Aluja (2012) shows, indeed, that work support acts as a significant buffer of work–family conflict especially for women. Also, Batt and Valcour (2003) found that having a supportive supervisor decreases women’s level of work–family conflict, but does not have this effect for men. Second, it is societally more accepted for women to utilize work–family benefits, as well as to rely on a wider range of organizational family support forms than men (Thompson and Cavallaro 2007). Managers and employees are also more likely to associate work-life conflict and flexible working more with female than with male employees (Smithson and Stokoe 2005). They may assume that working from home is more for family-friendly purposes for women compared to men, who they expect it to be used for performance enhancing purposes (Gerstel and Clawson 2014).

A supportive working context in which the proportion of colleagues working from home is high and the ideal worker culture absent, will be perceived more as a resource by telecommuting women, while due to the ‘gendered’ nature of family and household responsibilities and the utilization of work–family benefits, it arguably matters less for telecommuting men. Summarizing, we hypothesize that workplace support will buffer the negative effect of working from home on work–family conflict more strongly for women than for men (H4).

## Data and Methods

### Sample

We tested our hypotheses using the European Sustainable Workforce Survey (ESWS), a multi-actor organizational survey conducted within work establishments in Bulgaria, Finland, Germany, Hungary, the Netherlands, Portugal, Spain, Sweden, and the United Kingdom (Van der Lippe et al. 2016). A total of 259 work establishments, 869 work units, and 11,011 employees participated in the survey. Establishments were selected from six sectors (manufacturing, higher education, healthcare, IT and telecommunication, transport and logistics, and finance and banking sectors) and three size groups (20–99 workers, 100–250 workers, and 250 and more workers).

After the establishment (often the HR director) agreed to participate, employees, managers and the HR manager were addressed at work to participate in an online or paper-and-pencil questionnaire. In consultation with the organization, and where possible, we selected a number of work units that best represent the work establishment, and received a complete list of their workers who were invited to fill out a 20 min survey. The present analyses will mainly make use of the employee survey module. The response rate of employees was on average 61%, and it was almost complete (98%) among HR managers.^1^ Missing data on variables in the analyses (average 4.06% of answers missing across variables and 21.4% list-wise) were imputed using chained multiple imputations (m = 10).

### Measures

#### Dependent Variable

To measure *work*–*family conflict*, we used part of the SWING scale developed by Geurts et al. (2005) with the following three items: How often does it happen that (1) you do not have the energy to engage in leisure activities with your family or friends because of your job, (2) you have to work so hard that you do not have time for any of your hobbies, and (3) your work obligations make it difficult for you to feel relaxed at home, with answer categories from 1 never to 5 always. A higher score means more work–family conflict. The reliability of the scale is .86. We used regression factor scores obtained from maximum-likelihood factor analysis to construct a standardized variable for the analysis (descriptive statistics of constituting items and their factor loadings listed in “Appendix 1”).

#### Independent Variables

The questionnaire measured *individual employee’s working from home* by the relative frequency of working at home, following the measurement strategy recommended by Allen et al. (2015a). The survey included the following question: ‘In the past 12 months, how often have you worked at home during normal working hours? Exclude overtime.’ The response categories are (1) never or almost never, (2) less than 1 day a month, (3) less than 1 day a week, (4) 1 day a week, (5) 2 days a week, (6) 3 days a week, and (7) 4 or 5 days a week. We included this measure as a continuous variable by recoding the original question to the proportion of working hours per month spent working from home. The categories are never or almost never as 0 h, less than 1 day a month to 0.02, less than one day a week to 0.09, 1 day a week to 0.18, 2 days a week to 0.37, 3 days a week to 0.55, and 4–5 days a week to 0.83.^2^

To measure perception of *organizational and managerial work*–*family supportive culture* we used a reduced version of the work–family culture scale developed by Thompson et al. (1999). The scale measures three dimensions of supportive culture (managerial support, and two aspects of ideal worker culture: organizational demands that may interfere with family responsibilities, and negative consequences associated with devoting time to family responsibilities) with three items each on a 5-point Likert scale. Following the procedure of validation of the original paper, we conducted maximum-likelihood exploratory factor analyses with equamax rotation to verify the factor structure. Initial analyses with the complete set of nine items resulted in three factors above the eigenvalue of 1 corresponding to the three subscales, but the negative consequences subscale had very poor reliability (Cronbach’s alpha below .4) and two items from the scale were poorly explained by the factor structure (communality lower than .2, cf. Child 2006). We decided to exclude these two items and the repeated factor analysis retained two factors with eigenvalue higher than 1: the first factor representing managerial support and the second factor representing ideal worker culture. In order to allow a clear interpretation of the subscales, we further excluded an item from the ideal worker culture scale because it had loadings above the threshold of 3 (.35) on the managerial support factor, and the ratio between factor loadings was lower than the threshold of 2 (ratio = 1.5). The remaining items correlate adequately enough to suggest a factor structure (KMO = .7. Bartlett’s *p* = .000). The final factor analysis retained two factors above the eigenvalue of 1 and interpretable factor solution (see “Appendix 1”). Together, the two factors explained 47% of the total variance, with the first factor (managerial support) accounting for 32% and the second factor (ideal worker culture) explained an additional 15%. Both factors had an acceptable reliability (managerial support alpha = 0.77, ideal worker culture = 0.61). We used regression factor scores obtained from the exploratory factor analysis to construct a standardized variable for the analysis (descriptive statistics of constituting items and their factor loadings listed in “Appendix 1”).

To measure *working from home by team co*-*workers*, we counted the proportion of the respondent’s team co-workers (excluding the respondent) who work at home at least one day a month.

As we expect that men and women react differently to working from home, women (female employees = 1, and male employees = 0) is included as an independent variable as well.

#### Control Variables

We control for a number of variables indicated in the literature as being of influence on work–family conflict (cf. Allen et al. 2015a). At the employee level these include self-reported job autonomy (measured with 4 Likert-scale items listed in “Appendix 1”, alpha = 0.86), number of contract hours measured in hours, commuting time measured in minutes, and indicator variables measuring if the employee works on a flexible schedule, if the respondent has supervisory duties, if the respondent is higher educated (Bachelor’s degree and higher), and age and age squared. To control for family circumstances we added indicators of having a partner, the presence of minor children in the household, and number of hours per week spent on domestic work. To control for organizational characteristics, we added the size of the organization measured as the number of workers employed. Furthermore, we controlled for sector, including indicators for manufacturing, higher education, healthcare, telecommunication, banking and transport as dummies,^3^ as well as for country (Finland, Sweden, Germany, the Netherlands, the UK, Spain, Portugal, Hungary and Bulgaria).^4^ In the regression analyses, we squared root transformed hours spent on domestic work, commuting time and contracted hours at work, and took the logarithm of organizational size to normalize the distributions. Means and standard deviations of all variables are provided in Table 1.

**Table 1:** Sample descriptive statistics

	Mean	SD	Min.	Max.	Mean—women	Mean—men
Work-family conflict	7.04	2.87	3	15	7.11	6.95*
Working from home (proportion of month)	0.05	1.44	0	0.83	0.04	0.06*
Managerial support	10.72	2.38	3	15	10.66	10.80*
Ideal worker culture	8.11	2.39	3	15	7.99	8.27*
Proportion of co-workers working from home	0.18		0	1	0.16	0.20*
Female gender	0.56		0	1		
Supervisory position	0.19		0	1	0.15	0.25*
Job autonomy	8.98	3.34	4	20	8.98	8.98n.s.
Organizational tenure (years)	10.83	9.97	0.08	55	10.74	10.92n.s.
Flexible schedule (0/1)	0.42		0	1	0.38	0.47*
Contracted hours	36.92	7.96	0	60	35.62	38.57*
Commuting time	32.90	24.59	0	657	33.11	32.08*
Higher educated	0.51		0	1	0.54	0.48*
Age	42.27	11.06	14	81	42.08	42.49n.s.
Having a partner	0.73		0	1	0.72	0.76*
Having a young child	0.42		0	1	0.41	0.42n.s.
Hours of domestic work	11.55	10.10	0	168	13.35	9.18*
Organization size	736.47	1484.78	9	10,000	840.87	607.83*
Sector						
Manufacturing	0.23		0	1	0.15	0.32*
Health care	0.25		0	1	0.35	0.11*
Higher education	0.17		0	1	0.20	0.15*
Transport	0.13		0	1	0.09	0.19*
Financial services	0.12		0	1	0.14	0.10*
Telecommunication	0.09		0	1	0.07	0.13*
Country						
UK	0.07		0	1	0.07	0.07
Germany	0.09		0	1	0.09	0.09
Finland	0.07		0	1	0.08	0.06*
Sweden	0.10		0	1	0.09	0.11*
Netherlands	0.23		0	1	0.21	0.25*
Portugal	0.11		0	1	0.11	0.11
Spain	0.08		0	1	0.07	0.10*
Hungary	0.12		0	1	0.13	0.12
Bulgaria	0.13		0	1	0.16	0.10*
Paper-and-pencil mode	0.29		0	1	0.30	0.26*

Means and standard deviations reported using the non-imputed dataset and all available information per variable. N = 11,011. Work-family conflict, managerial support, organizational demands and job autonomy scales are sums of constituting items and are reported for descriptive purposes. The regression analyses contain standardized factor scores to measure these scales. Last column lists independent sample *t* test results, *significant at least at the 95% level (two-sided)

### Analytical Strategy

We used a three-level multilevel random intercept regression models to analyze the relation between working from home and work–family conflict, as we have employees nested in teams in organizations. All models include random intercepts for organization and team level, as well as an individual-level error term, to adequately model error variance at the three levels of the survey. Initial analysis without any predictors indicated that there is significant variation in work–family conflict at the organization and team level (19.3% of the variance is at organization level and 14.4% of the variance is at work unit level), confirming the need for random intercept models.

Model 1 includes working from home and the control variables. Model 2 adds managerial support, ideal worker culture, and co-worker working from home. Model 3 and 4 adds the interactions with these variables and working from home, and Model 5 adds the interactions of gender with working from home. Model 6 combines all interactions in one model. Finally, the full model is analyzed separately for men and women to see if work–family culture and co-worker behavior buffers the relation between working from home and work–family conflict in different ways.^5^ Finally, additional models (reported in the online Appendix Table A1, for consideration of space) formally test the three-way interaction between gender, working from home and organizational support.

## Results

Women participating in the survey experience a slightly higher amount of work–family conflict, but men spend a slightly higher proportion of their regular working time working from home than women do and more often have flexible schedules (see descriptive statistics in Table 1). This latter finding appears to be due to sectoral effects: more than one third of women in the sample are working in health care—compared to only around ten percent of men—where working from home and flexible starting and finishing times are less prevalent. We see furthermore that men work longer hours and that women spend more time on domestic work. Perceptions of managerial support do not differ much between men and women, but men perceive more an ideal worker culture. Moreover, Table 1 shows that women are more found than men in larger organizations.

Table 2 presents the results regarding the relation between working from home—measured as the proportion of working time that employees spend working from home—and work–family conflict, and how this relation varies by perceptions of a supportive organizational culture. Model 1 shows that working from home is associated with higher levels of work–family conflict, supporting our first hypothesis, although the effect is rather weak: 10% (around one standard deviation) increase in the proportion of time devoted to working from home results in 0.05 standard deviation increase in work–family conflict, providing support for H1. Model 2 adds the organizational support factors to the models: as expected, perceived managerial support decreases and ideal worker culture increase work–family conflict. The positive effect of working from home remains. Model 3 to 4 adds the interaction of working from home with organizational context factors (managerial support, ideal worker culture, and co-worker engagement); the one-for-one test of interactions do not indicate that the organizational context moderates the impact of working from home on work–family conflict. Model 5 studies if the relation between working from home and work–family conflict is different for men and women, and results show that working from home increases work–family conflict more for women than for men. In fact, the effect of working from home among men does not reach statistical significance, whilst it significantly increases work–family conflict for women. Model 6 includes all interactions: the interaction between ideal worker culture and working from home becomes positive significant, meaning that working from home is more detrimental for work-life conflict in organizations with an ideal worker culture, providing support for H2b. The hypotheses on the moderating effects of managerial support (H2a) and co-worker engagement in working from home (H2c) are not supported in the completed model either, while the gender and working from home interaction remains significant, providing support for H3. The full model explains 51% of the total variance—after accounting for country, sector, organizational size, and survey mode effects—in work–family conflict across organizations and 40% of the variance across teams. Supportive organizational culture, working from home and their interactions explain 47% of organizational variance unexplained by control variables, and 25% of the team variance, showing that the factors we consider are relevant for understanding the varying level of work–family conflict experienced by workers across organizations.

**Table 2:** Hierarchical linear regression analyses of work–family conflict on working from home

	Model 1	Model 2	Model 3	Model 4	Model 5	Model 6	Model 6 female	Model 6 male
Working from home (proportion of working month × 10)	0.05*** (0.01)	0.03*** (0.01)	0.03*** (0.01)	0.04*** (0.01)	0.01^†^ (0.01)	0.02 (0.01)	0.08*** (0.02)	0.01 (0.01)
Managerial support		− 0.17*** (0.01)	− 0.17*** (0.01)	− 0.17*** (0.01)	− 0.17*** (0.01)	− 0.17*** (0.01)	− 0.17*** (0.01)	− 0.18*** (0.02)
Ideal worker culture		0.30*** (0.01)	0.30*** (0.01)	0.30*** (0.01)	0.30*** (0.01)	0.29*** (0.01)	0.29*** (0.01)	0.30*** (0.02)
Proportion of co-workers working from home		0.01 (0.01)	0.01 (0.01)	0.01 (0.01)	0.01 (0.01)	0.01 (0.01)	0.03*** (0.01)	− 0.01 (0.01)
Working from home × Managerial support			0.00 (0.01)			0.01 (0.01)	− 0.00 (0.01)	0.02^†^ (0.01)
Working from home × Ideal worker culture			0.01^†^ (0.01)			0.01* (0.01)	0.01 (0.01)	0.02^†^ (0.01)
Working from home × Pr. co-workers WFH				− 0.00 (0.00)		− 0.00 (0.00)	− 0.01* (0.00)	0.00 (0.00)
Working from home × Female					0.04*** (0.01)	0.04*** (0.01)		
Female gender	0.04^†^ (0.02)	0.04* (0.02)	0.04* (0.02)	0.04* (0.02)	0.02 (0.02)	0.02 (0.02)		
Supervisory position	0.20*** (0.02)	0.17*** (0.02)	0.17*** (0.02)	0.17*** (0.02)	0.17*** (0.02)	0.17*** (0.02)	0.19*** (0.03)	0.16*** (0.03)
Job autonomy	0.15*** (0.01)	0.07*** (0.01)	0.07*** (0.01)	0.07*** (0.01)	0.07*** (0.01)	0.07*** (0.01)	0.06*** (0.01)	0.10*** (0.02)
Organizational tenure (years)	0.00*** (0.00)	0.00 (0.00)	0.00 (0.00)	0.00 (0.00)	0.00 (0.00)	0.00 (0.00)	− 0.00 (0.00)	0.00*** (0.00)
Flexible schedule (0/1)	− 0.00 (0.02)	0.00 (0.02)	0.00 (0.02)	0.00 (0.02)	0.00 (0.02)	0.00 (0.02)	− 0.02 (0.03)	0.01 (0.03)
Contracted hours (sqwrt)	0.14*** (0.01)	0.12*** (0.01)	0.12*** (0.01)	0.12*** (0.01)	0.12*** (0.01)	0.12*** (0.01)	0.11*** (0.02)	0.12*** (0.02)
Commuting time (sqrt)	0.04*** (0.01)	0.04*** (0.00)	0.04*** (0.00)	0.04*** (0.00)	0.04*** (0.00)	0.04*** (0.00)	0.04*** (0.01)	0.04*** (0.01)
Higher educated	0.07*** (0.02)	0.03 (0.02)	0.03 (0.02)	0.03 (0.02)	0.03 (0.02)	0.03 (0.02)	0.03 (0.03)	0.04 (0.03)
Age	0.02*** (0.01)	0.02*** (0.01)	0.02*** (0.01)	0.02*** (0.01)	0.02*** (0.01)	0.02* (0.01)	0.02* (0.01)	0.01 (0.01)
Age^2^	− 0.00*** (0.00)	− 0.00*** (0.00)	− 0.00*** (0.00)	− 0.00*** (0.00)	− 0.00*** (0.00)	− 0.00*** (0.00)	− 0.00* (0.00)	− 0.00^†^ (0.00)
Having a partner	0.06*** (0.02)	0.06*** (0.02)	0.06*** (0.02)	0.06*** (0.02)	0.06*** (0.02)	0.06*** (0.02)	0.05^†^ (0.03)	0.08* (0.03)
Having a young child	− 0.00 (0.02)	0.00 (0.02)	0.00 (0.02)	0.00 (0.02)	0.00 (0.02)	0.00 (0.02)	− 0.03 (0.03)	0.04 (0.03)
Hours of domestic work (sqrt)	0.04*** (0.01)	0.03*** (0.01)	0.03*** (0.01)	0.03*** (0.01)	0.03*** (0.01)	0.03*** (0.01)	0.05*** (0.01)	0.01 (0.01)
Organization size (log)	− 0.01 (0.01)	− 0.01 (0.01)	− 0.01 (0.01)	− 0.01 (0.01)	− 0.01 (0.01)	− 0.01 (0.01)	0.01 (0.01)	− 0.024 (0.02)
Intercept	− 1.77*** (0.18)	− 1.56*** (0.16)	− 1.56*** (0.16)	− 1.57*** (0.16)	− 1.54*** (0.16)	− 1.54*** (0.16)	− 1.75*** (0.21)	− 1.11*** (0.24)
σ(organization)	0.19*** (0.02)	0.14*** (0.02)	0.14*** (0.02)	0.14*** (0.02)	0.14*** (0.02)	0.14*** (0.02)	0.11*** (0.02)	0.18*** (0.02)
σ(work unit)	0.17*** (0.02)	0.15*** (0.01)	0.15*** (0.01)	0.15*** (0.01)	0.15*** (0.01)	0.15*** (0.01)	0.17*** (0.02)	0.11*** (0.04)
σ(Residual)	0.85*** (0.01)	0.80*** (0.01)	0.80*** (0.01)	0.80*** (0.01)	0.80*** (0.01)	0.80*** (0.01)	0.80*** (0.01)	0.80*** (0.01)
Number of employees	11,011	11,011	11,011	11,011	11,011	11,011	6069	4751
Number of work units	868	868	868	868	868	868	781	759
Number of organizations	257	257	257	257	257	257	256	252

Estimated on multiple imputed data (M = 10). Standard errors corrected for multiple imputation bias using Rubin’s rules and are in parentheses. Models include indicators for country, sector, and survey mode (not shown). Source: ESWS

^†^*p* < 0.10; *p < 0.05; **p < 0.01; ***p < 0.01 (2-sided)

Model 6 is estimated for men and women separately^6^ to investigate gender differences. We also estimated a pooled model including three-way interactions between working from home, organizational context variables, and gender to formally test H4 (full results available in online Appendix Table A1, Model 7) as well as a less parsimonious full gender interaction model (results available in online Appendix Table A1, Model 8). The results of model 6 show clearly different patterns for men and women: ideal worker culture and managerial support increase the positive relation between working from home and work–family conflict but only for men and the findings are only significant on the .1 level (2-sided). When formally tested, the three-way interaction between gender, managerial support and managerial support is significant on the .1 level (2-sided) (b = − .02, SE = .01, *p* = .08). In the full interaction model, however, the effect is of similar size, but not significant on the .1 level (2-sided) (b = − .02, SE = .01, *p* = .12). These results provide, although modest, support for H4 that working from home is more beneficial in combination with managerial work-life support for women than for men with regards to less work-life conflict. In addition, if more co-workers work from home own working from home is less negatively related to work–family conflict among women, but this is not the case for men. Again, when formally tested, the negative moderating effect of co-worker engagement in working from home is stronger among women, although only significant on the .1 level (2-sided) (b = − .01, SE = .00, *p* = 0.06), but both in the pooled and full interaction models. This result is also in line with H4.

## Conclusion

Empirical research on the influence of working from home on the work–family interface does not produce convincing outcomes regarding a decrease of work–family conflict. In this contribution we argued that working from home will lead to more facilitation of work and family life, if a supportive work context is available. Specifically, managerial support, ideal worker culture, as well as the number of colleagues working from home are expected to moderate the relation between working from home and work–family conflict. Gender differences are explored therein. Using unique 2016 data from 11,011 employees in 869 teams in 259 work establishments in 9 European countries, we tested several hypotheses. Our multi-level approach resulted in four substantive findings.

The first conclusion is that elements of the work context help to explain how working from home can alleviate or increase work–family conflict for both men and women. We studied under what conditions working from home leads to work–family conflict, and conclude that if an ideal worker culture exists, where the norm is to work hard, to work overtime, and to take work home at night or in the weekend to get ahead in the organization, working from home will lead to more work–family conflict. This implies that in organizations where no such culture exists, results in less work–family conflict. This is in line with research from Kelly et al. (2010) who also showed the existence of the ideal worker culture. Working part-time which alleviates the time pressure, is no option in these organizations (Rose et al. 2013). However, we didn’t find that the perception of a supportive manager is important as well for work–family conflict. It could be that the backbone of work–family conflict is an ideal worker culture, which creates demands that interfere with family responsibilities. The original research by Thompson et al. (1999) already showed more influence of the ideal worker culture than managerial support on work–family conflict. However, Anderson et al. (2002) reported managerial support to be more important than the ideal worker culture. Differences could be due to the samples used. Anderson et al. made use of a large scale sample of employed adults, not necessarily nested in organizations.

The second conclusion is that the relation between working from home and work–family conflict is clearly gendered. Working from home leads to more work–family conflict for women. The fact that they are working from home makes the boundaries between work–and family life permeable which increases the chance on work–family interference especially for women (see also, Kim in this issue; Kurowska in this issue). This is in line with many research findings on related topics showing that the work and family domain are more linked for women than men (Van der Lippe 2007; Van der Horst et al. 2014). When studying ambitions of men and women, women more often take the linkage between work and family life into account, whereas men are more fully concentrated on their work career (Van der Horst et al. 2014); looking at work–family conflict, women also more often report to be influenced by their family obligations and men by work obligations (Van der Lippe et al. 2006). Our finding adds to the extant literature on gender differences between experiencing work and family life.

The third conclusion is that the work context appears to work differently for men than for women. Having many co-workers working home alleviates some of the influence of working from home on work–family conflict for women, but men are not influenced by this. This type of support may also especially help women, as they experience more stress in general than men (Matud 2004). One could imagine that they are more sensitive to this behavior by co-workers and are more likely to benefit from it. Co-workers working from home are more likely to be understanding of the potential conflicting situations which helps women more than men. Note that this conclusion is only true for workplaces where and jobs for which it is possible at all to work from home (so for example not for nurses). Unexpectedly, although only marginally significant, managerial support increases the positive relation between working from home and work–family conflict for men but not for women. The influence of ideal worker culture seems to be mainly driven mainly by male respondents, although the differences between men and women are very slight. Norms in organizations might still dictate a full commitment of men to work (Van der Lippe 1994; Treas and Drobnic 2010), which heightens the experiences of work–family conflict. This has perhaps also to do with the feminity stigma (Williams et al. 2013; Chung in this issue), where men may face a double stigma when using flexible working arrangements due to the fact that such arrangements deviate away from the ideal worker norm but also from masculinity. For women, work ethic is also positively associated with their job, but only if women’s gender role values are taken into account, which negatively relate to women’s work (Stam et al. 2014).

The fourth conclusion is that it is important to take a multi-level approach to this issue. Our contribution showed the importance of incorporating the team level and organizational context to understand why working from home is related to work–family conflict, and why this works differently for men and women. Not having information on the teams and organizations where these men and women work would make it possible to describe that women experience more work–family conflict when working from home, but not what the role of colleagues in the team and the organization thereby is.

We recognize that there are also limitations to the research presented here. First and foremost, it is likely that there is selection of men and women who experience work–family conflict, and maybe they are selected into organizations that provide working from home policies. There might even be a possibility of reverse causation: organizations in which men and women experience comparatively more work–family conflict may like to work from home. We have tried to take this partly into account by controlling for a number of family responsibilities and organizational factors. This gives us confidence with respect to our research strategy, but nevertheless, we encourage new research using a long time panel set up, in which organizations and their employees are followed over some time to better unravel the causes and consequences of work–family conflict. Second, we have focused in this study on the experienced work–family culture in the organization by the employee. Although we still argue this to be important, we can imagine that focusing on the actual behavior (and the gender) of the manager of the employee, will contribute to understanding the influence of working from home and work–family conflict better (Hammer et al. 2009; Matthews et al. 2013). Third, work–family conflict is a subjective feeling, and we encourage other researchers to also study more behavioral consequences such as time spent on parenting, time spent on housework, the amount of leisure activities, and having contacts with family and friends, which may be all behavioral outcomes of work–family conflict. However, we moved beyond existing work–family conflict scales by also including leisure time as a potential source of conflict.

Our results leave several other questions unanswered, such as what happens with men when they work from home. Managerial support and organizational demands both pointed in the direction of having more work–family conflict, but it is unclear what is actually happening. We therefore propose qualitative research where the researcher studies processes of support and the culture in one or two organizations for a longer period of time. Also reactions of the team when working from home as a male employee are important to take into account. Another way to go would be to study more closely which tasks are performed at home when working from home. Tasks which apparently lead to more work–family conflict, than when performed at the office. Moreover, it would be informative to study why people work from home, is it to reduce work–family conflict as is the assumption in this paper, or also to save commuting time (Bailey and Kurland 2002). All in all, our study showed working from home leads to more work–family conflict, especially when workers perceive an ideal worker culture at their workplace. Women experience more work–family conflict from working home than men, but the more colleagues are working from home the less conflict these women experience.

### Electronic supplementary material

Below is the link to the electronic supplementary material.

Supplementary material 1 (PDF 132 kb)

Supplementary material 2 (PDF 158 kb)

## Appendix 1: Scale Items and Factor Loadings

**Table:**

Scale	Item	Mean	STD	Factor loading
Work–family conflict	How often does it happen that… You do not have the energy to engage in leisure activities with your family or friends because of your job?	2.51	1.07	0.83
	You have to work so hard that you do not have time for any of your hobbies?	2.25	1.11	0.85
	Your work obligations make it difficult for you to feel relaxed at home?	2.28	1.09	0.77
Managerial support	My manager is understanding when I have to put my family first	3.89	0.95	0.82
	Higher management encourages supervisors to be sensitive to employees’ family concerns	3.11	0.96	0.51
	My manager is very accommodating of family-friendly needs	3.72	0.96	0.83
Ideal worker culture	Employees are often expected to take work home at night or in the weekend	2.37	1.10	0.51
	To turn down a promotion or transfer for family-related reasons will seriously hurt one’s career progress in this organization	2.84	0.94	0.45
	To get ahead in this organization, employees are expected to work overtime	2.89	1.14	0.81
Job autonomy	How often are you free to decide … The tasks you do in your job	2.45	1.02	0.74
	How you do your work	2.03	0.90	0.80
	The order in which you carry out tasks	2.04	0.92	0.82
	When you do your work	2.44	1.13	0.77

Means and standard deviations reported using the non-imputed dataset and all available information per variable. N = 11,011. All items are measured on Likert-type scales ranging between 1 and 5. Work-family conflict and job autonomy factor loadings are estimated separately, and managerial support and organizational demands factor loadings are estimated jointly using equamax rotation. Joint exploratory factor analyses of all items with equamax rotation confirmed the 4-factor solution, with loadings highly similar to the ones in the separate factor analyses

## Footnotes

1 The response rate differed across organizations and teams. We conducted additional analyses to investigate whether variation in response rates in any way biases our results. We split the sample to teams with high and low response rate, choosing 80% as a cut-off point and repeated the analysis of the full model (Model 6) in Table 2. In addition, we also compared the results from the full model in Table 2 with a regression weighted by the team-level response rate. In essence, the analysis with weights addresses the problem of response biasing the regression results by weighting down the influence of teams with low response rate. Neither of these comparisons indicate that there is any bias due to response.

2 We calculated with 173 working hours per month, and 4 h in case the response was less than 1 day.

3 While the prevalence of working from home differs across economic sectors, additional models including interaction between working from home and sector do not reveal differences in the way working from home affects work-family conflict. Results from these models are available in the online Appendix (Table A2, Model 9).

4 We estimated additional models including a three-way interaction terms between working from home, organizational support and country to investigate country-differences in the hypothesized moderating effect of supportive organizational culture. The results do not show country variation. Results from these models are available in the online Appendix (Table A2, Model 10).

5 The organizational and team level variance component estimates in intercept-only and full models did not statistically differ between men and women.

6 In order to ensure that we measure the same constructs in the male and female subsamples, we performed additional multi-group confirmatory factor analysis to test the measurement equivalence of our scales by gender. The results of factor loadings and intercept equivalence model show that a loading of a single item on the autonomy scale differ between men and women. The partially constrained model shows an equally good fit as the unconstrained model (ΔLR = 4.87, p = 0.77), indicating full measurement invariance on the work-family conflict and organizational work-family culture scales, and partial equivalence on the control variable autonomy scale.
