# Supplementary material for: Beyond Formal Access: Organizational Context, Working From Home, and Work–Family Conflict of Men and Women in European Workplaces
Source: Soc Indic Res. 2018 Oct 5;151(2):383–402. doi: 10.1007/s11205-018-1993-1 (PMC7505867; doi:10.1007/s11205-018-1993-1)
Supplement: Supplementary file 1 — Supplementary material 1 (PDF 132 kb) [file 11205_2018_1993_MOESM1_ESM.pdf]

Table A1. Hierarchical linear regression analyses of work-family conflict on working from home - partial and full gender interaction model

	Model7	Model8
Working from home (proportion of working month *10)	0.01	0.004
	(0.01)	(0.01)
Managerial support	-0.18***	-0.18***
	(0.02)	(0.02)
Ideal worker culture	0.3***	0.29***
	(0.02)	(0.02)
Proportion of co-workers working from home	0.01	-0.01
	(0.01)	(0.01)
Working from home × Mangerial support	0.01+	0.01+
	(0.01)	(0.01)
Working from home × Ideal worker culture	0.02+	0.01+
	(0.01)	(0.01)
Working from home × proportion of co-workers WFH	0.001	0.002
	(0.003)	(0.003)
Working from home × Female	0.07***	0.08***
	(-0.02)	(-0.02)
Female gender	-0.003	-0.622*
	(0.03)	(0.296)
Supervisory position	0.17***	0.16***
	(0.02)	(0.03)
Job autonomy	0.07***	0.095***
	(0.01)	(0.01)
Organizational tenure (years)	0.002	0.004*
	(0.001)	(0.002)
Fle×ible schedule (0/1)	0.003	0.021
	(0.02)	(0.03)
Contracted hours (sqrt)	0.12***	0.12***
	(0.01)	(0.02)
Commuting time (sqrt)	0.04***	0.04***
	(0.005)	(0.007)
Higher educated	0.03	0.04
	(-0.02)	(-0.03)
Age	0.02*	0.01
	(0.01)	(0.01)
Age2	-0.0002***	-0.0001
	(0.00007)	(0.0001)
Having a partner	0.06***	0.07*
	(0.02)	(0.03)
Having a young child	0.001	0.039
	(0.02)	(0.03)
Hours of domestic work (sqrt)	0.03***	0.01
	(0.01)	(0.01)
Organization size (log)	-0.01	-0.02
	(0.01)	(0.01)
Managerial support × Female	0.02	0.01
	(0.02)	(0.02)
Ideal worker culture × Female	-0.01	0.001

	Model7	Model8
	(-0.02)	(-0.02)
Proportion of co-workers working from home × Female	0.01	0.04***
	(0.01)	(0.01)
Working from home × Mangerial support × Female	-0.02+	-0.02+
	(0.01)	(0.01)
Working from home × Ideal worker culture × Female	-0.01	-0.004
	(0.01)	(0.01)
Working from home × Pr. co-workers working from home × Female	-0.01+	-0.01*
	(0.005)	(0.005)
Autonomy × Female		-0.04+
		(0.02)
Higher educated × Female		-0.02
		(0.04)
Commuting time (sqrt) × Female		-0.0001
		(0.01)
Fle×ible schedule (0/1) × Female		-0.04
		(0.04)
Contracted hours (sqrt) × Female		-0.01
		(0.02)
Supervisory position × Female		0.03
		(0.04)
Organizational tenure (years) × Female		-0.004*
		(0.002)
Age × Female		0.01
		(0.01)
Age2 × Female		0.000
		(0.001)
Having a partner × Female		-0.02
		(0.04)
Having a young child × Female		-0.06
		(0.04)
Hours of domestic work (sqrt) × Female		0.04*
		(0.01)
Organization size (log) × Female		0.03*
		(0.01)
Intercept	-1.53***	-1.12***
	(0.16)	(0.23)
σ(organization)	0.14***	0.14***
	(0.02)	(0.02)
σ(work unit)	0.15***	0.14***
	(0.01)	(0.01)
σ(Residual)	0.8***	0.8***
	(0.01)	(0.01)
Number of employees	11011	11011
Number of work units	868	868
Number of organizations	257	257

Notes: † p<0.10 * p<0.05 ** p<0.01 *** p<0.01 (2-sided). Estimated on multiple imputed data (M=10). Standard errors corrected. Models include indicators for country sector and survey mode, interacted with gender (Model 8). Source: ESWS
