# Supplementary material for: Beyond Formal Access: Organizational Context, Working From Home, and Work–Family Conflict of Men and Women in European Workplaces
Source: Soc Indic Res. 2018 Oct 5;151(2):383–402. doi: 10.1007/s11205-018-1993-1 (PMC7505867; doi:10.1007/s11205-018-1993-1)
Supplement: Supplementary file 2 — Supplementary material 2 (PDF 158 kb) [file 11205_2018_1993_MOESM2_ESM.pdf]

Table A2. Hierarchical linear regression analyses of work-family conflict on working from home - sector and country moderation models

	Model 9	Model 10
Working from home (proportion of working month *10)	0.04***	0.09*
	(0.02)	(0.04)
sector (ref: Manufacturing)		
Health care	0.05	0.12***
	(0.05)	(0.05)
Higher Education	-0.003	0.01
	(0.06)	(0.05)
Transport	-0.03	-0.02
	(0.06)	(0.05)
Financial Services	-0.07	-0.07
	(0.06)	(0.05)
Telecommunication	-0.07	-0.01
	(0.06)	(0.06)
Health care × Working from home	0.01	
	(0.02)	
Higher Education × Working from home	0.03	
	(0.02)	
Transport × Working from home	-0.03	
	(0.03)	
Financial Services × Working from home	-0.01	
	(0.03)	
Telecommunication × Working from home	-0.02	
	(0.02)	
Female gender	0.04+	0.04*
	(0.02)	(0.02)
Supervisory position	0.19***	0.17***
	(0.02)	(0.02)
Job autonomy	0.15***	0.07***
	(0.01)	(0.01)
Organizational tenure (years)	0.003***	0.002
	(0.001)	(0.001)
Fle×ible schedule (0/1)	0.001	-0.0002
	(0.022)	(0.021)
Contracted hours (sqrt)	0.14***	0.12***
	(0.01)	(0.01)
Commuting time (sqrt)	0.04***	0.04***
	(0.005)	(0.005)
Higher educated	0.06***	0.03
	(0.02)	(0.02)
Age	0.02***	0.02*
	(0.01)	(0.01)
Age2	-0.0002***	-0.0002***
	(0.00008)	(0.00007)
Having a partner	0.06***	0.06***
	(0.02)	(0.02)
Having a young child	-0.0007	0.00082
	(0.023)	(0.022)
Hours of domestic work (sqrt)	0.04***	0.03***
	(0.01)	(0.01)
Organization size (log)	-0.01	-0.006
	(0.01)	(0.01)
country (ref: United Kingdom)		
Germany	0.28***	0.25***
	(0.08)	(0.1)
Finland	-0.1	-0.14
	(0.09)	(0.11)

	Model 9	Model 10
Sweden	-0.02	0.06
	(0.08)	(0.09)
Netherlands	0.03	-0.05
	(0.07)	(0.08)
Portugal	0.06	0.09
	(0.08)	(0.09)
Spain	0.02	0.02
	(0.09)	(0.1)
Hungary	0.12	0.24***
	(0.08)	(0.09)
Bulgaria	0.31***	0.42***
	(0.08)	(0.09)
Managerial support		-0.09*
		(0.04)
Ideal worker culture		0.39***
		(0.04)
Proportion of co-workers working from home		0.001
		(0.03)
Working from home × Mangerial support		0.002
		(0.03)
Working from home × Ideal worker culture		0.01
		(0.02)
Working from home × Proportion of co-workers working from home		-0.01
		(0.01)
Germany × Working from home		-0.06
		(0.06)
Finland × Working from home		-0.04
		(0.05)
Sweden × Working from home		-0.05
		(0.06)
Netherlands × Working from home		-0.03
		(0.05)
Portugal × Working from home		-0.04
		(0.05)
Spain × Working from home		-0.05
		(0.05)
Hungary × Working from home		-0.09*
		(0.05)
Bulgaria × Working from home		-0.08
		(0.06)
Germany × Managerial support		-0.09
		(0.05)
Finland × Managerial support		-0.02
		(0.06)
Sweden × Managerial support		-0.09+
		(0.05)
Netherlands × Managerial support		-0.09*
		(0.05)
Portugal × Managerial support		-0.04
		(0.05)
Spain × Managerial support		-0.06
		(0.05)
Hungary × Managerial support		-0.18***
		(0.05)
Bulgaria × Managerial support		-0.09+
		(0.05)
Germany × Ideal worker culture		-0.06
		(0.06)

	Model 9	Model 10
Finland × Ideal worker culture		-0.11+
		(0.06)
Sweden × Ideal worker culture		-0.15***
		(0.05)
Netherlands × Ideal worker culture		-0.09+
		(0.05)
Portugal × Ideal worker culture		-0.15***
		(0.05)
Spain × Ideal worker culture		-0.12*
		(0.05)
Hungary × Ideal worker culture		-0.1+
		(0.05)
Bulgaria × Ideal worker culture		-0.08
		(0.05)
Germany × Pr. of co-workers working from home		0.01
		(0.05)
Finland × Pr. of co-workers working from home		0.03
		(0.04)
Sweden × Pr. of co-workers working from home		0.01
		(0.04)
Netherlands × Pr. of co-workers working from home		0.03
		(0.03)
Portugal × Pr. of co-workers working from home		0.01
		(0.04)
Spain × Pr. of co-workers working from home		0.01
		(0.05)
Hungary × Pr. of co-workers working from home		0.003
		(0.04)
Bulgaria × Pr. of co-workers working from home		-0.03
		(0.05)
Germany × Working from home × Mangerial support		0.05
		(0.04)
Finland × Working from home × Mangerial support		-0.01
		(0.03)
Sweden × Working from home × Mangerial support		0.002
		(0.04)
Netherlands × Working from home × Mangerial support		0.01
		(0.03)
Portugal × Working from home × Mangerial support		-0.02
		(0.03)
Spain × Working from home × Mangerial support		-0.02
		(0.03)
Hungary × Working from home × Mangerial support		0.04
		(0.03)
Bulgaria × Working from home × Mangerial support		-0.01
		(0.03)
Germany × Working from home × Ideal worker culture		-0.01
		(0.03)
Finland × Working from home × Ideal worker culture		0.01
		(0.03)
Sweden × Working from home × Ideal worker culture		0.08*
		(0.04)
Netherlands × Working from home × Ideal worker culture		0.01
		(0.03)
Portugal × Working from home × Ideal worker culture		-0.01
		(0.03)
Spain × Working from home × Ideal worker culture		-0.02
		(0.03)

	Model 9	Model 10
Hungary × Working from home × Ideal worker culture		-0.01
		(0.03)
Bulgaria × Working from home × Ideal worker culture		0.002
		(0.03)
Germany × Working from home × Pr. co-workers working from home		0.01
		(0.01)
Finland × Working from home × Pr. co-workers working from home		0.01
		(0.01)
Sweden × Working from home × Pr. co-workers working from home		-0.002
		(0.01)
Netherlands × Working from home × Pr. co-workers working from home		0.004
		(0.01)
Portugal × Working from home × Pr. co-workers working from home		0.01
		(0.01)
Spain × Working from home × Pr. co-workers working from home		0.01
		(0.01)
Hungary × Working from home × Pr. co-workers working from home		0.02+
		(0.01)
Bulgaria × Working from home × Pr. co-workers working from home		0.02
		(0.02)
Intercept	-1.77***	-1.55***
	(0.18)	(0.16)
σ(organization)	0.03***	0.14***
	(0.02)	(0.02)
σ(β(working from home[organization]))	0.19***	
	(0.02)	
σ(work unit)	0.17***	0.15***
	(0.02)	(0.01)
σ(Residual)	0.85***	0.8***
	(0.01)	(0.01)
Number of employees	11011	11011
Number of work units	868	868
Number of organizations	257	257

Notes:† p<0.10 * p<0.05 ** p<0.01 *** p<0.01 (2-sided). Estimated on multiple imputed data (M=10). Standard errors corrected for multiple imputation bias using Rubin's rules and are in parentheses. Models include indicators for survey mode. Source: ESWS
